# Melatonin‐Producing Microorganisms: A Rising Research Interest in Their Melatonin Biosynthesis and Effects on Crops

**DOI:** 10.1111/jpi.70081

**Published:** 2025-09-22

**Authors:** Sang‐Mo Kang, Ashim Kumar Das, Da‐Sol Lee, Byung‐Wook Yun, Marino B. Arnao, In‐Jung Lee

**Affiliations:** ^1^ Department of Applied Biosciences Kyungpook National University Daegu Republic of Korea; ^2^ Department of Plant Biology (Plant Physiology), Faculty of Biology University of Murcia Murcia Spain

**Keywords:** algae, *E. coli*, melatonin, phytomelatonin, plant pathogen defense, plant‐microbe, rhizobiome, *Saccharomyces*, stress responses

## Abstract

Melatonin is imperative in animals and plants, contributing to multiple physiological roles, and its microbial production could offer an eco‐friendly alternative to synthetic melatonin. However, detecting and characterizing it in microorganisms remains ongoing, and the biosynthesis pathways are still poorly explored. We noted that not all microorganisms possess similar enzymes and substrates for melatonin production. Its biosynthesis pathway is well‐characterized in yeast, potentiating its importance in agricultural practices in a melatonin‐dependent manner. Intercellular melatonin production in algae and fungi boosts their resilience to oxidative cell death by activating the antioxidant defenses. Few studies on the use of *Bacillus sp*., *Pseudomonas sp*., and *Enterobacter sp*. have shown that these bacteria increase their endogenous melatonin contents, which may exchange with their host plants; thereby, mitigating abiotic stresses by modulating cellular damages, ion exchanges, hormonal levels, and related transcript expressions. Though plant‐growth‐promoting microbes show promise to enhance crop production, melatonin‐producing microorganisms (M‐PMs) are limited in identification, and their ecological and biological applications are still underutilized in agriculture. With the compounded benefits from M‐PMs, it could be an untapped tool for rhizospheric bioengineering. Therefore, this review delivers comprehensive insights into M‐PMs for practicing sustainable agriculture under increased climatic changes.

**Abbreviations**
AADC: aromatic l‐amino acid decarboxylase
AANAT: arylalkylamine N‐acetyltransferase
APA: American Psychiatric Association
ASMT: N‐acetylserotonin methyltransferase
cAMP: cyclic adenosine monophosphate
COMT: caffeic acid O‐methyltransferase
DDC: 5‐hydroxy‐l‐tryptophan decarboxylase
DHPR: dihydropteridine reductase
DW: dry weight
EcRiml: *E. coil* RimI
FW: fresh weight
GNA1: glucosamine‐6‐phosphate N‐acetyltransferase
GPCRs: G protein‐coupled receptors
GPR‐3: G protein‐coupled receptor 3
HIOMT: hydroxyindole‐O‐methyltransferase
HPA2: histone acetyltransferase
IAT4: putative indolamine N‐acetyltransferase
MAPK: mitogen‐activated protein kinases
M‐PMs: melatonin‐producing microorganisms
ND: not detected
NM: not mentioned
P4H: phenylalanine 4‐hydroxylase
PAA1: polyamine acetyltransferase
PCBD1: pterin‐4‐alpha‐carbinolamine dehydratase (4a‐hydroxytetrahydrobiopterin dehydratase)
PGPMs: plant growth‐promoting microorganisms
PGPR: plant‐growth‐promoting rhizobacteria
PTS: 6‐pyruvoyl‐tetrahydropterin synthase
ROS: reactive oxygen species
SNAT: serotonin N‐acetyltransferase
SPR: sepiapterin reductase
T5H: tryptamine 5‐hydroxylase
TDC: tryptophan decarboxylase
TPH: tryptophan hydroxylase
USD: United States dollar

## 1 Introduction

Melatonin, first identified in animals in 1958 [1, 2], in plants in 1995 [3, 4], and in microbes (particularly in yeasts) in 1999 [5], has since been recognized as an excellent prospect in the drug industry and agriculture. In general, melatonin is used for dietary purposes that aid as a sleep supplement, tranquilizer, antioxidant, and antiaging component [6], making it a versatile tool for human health [7]. Recently, the American Psychological Association (APA), according to the National Sleep Foundation (NSF) reported that at least 40 million Americans suffer from over 70 different sleep disorders, and 60 percent of adults report having sleep problems a few nights a week or more. In addition, more than 40 percent of adults experience daytime sleepiness severe enough to interfere with their daily activities at least a few days each month. Furthermore, 69 percent of children experience one or more sleep problems for a few nights or more during a week [8, 9]. Insomnia has notably increased after the COVID‐19 pandemic [10], which intensified the demand for commercial melatonin. Consequently, the global melatonin market value reached 4000 tons in 2019 with a total worth of USD 1.3 billion [11], and is expected to increase more [8]. Nonetheless, commercial melatonin totally depends on synthetic chemicals, neither a sustainable nor an eco‐friendly process.

Recent investigations and advanced technologies are forwarding the melatonin production from microorganisms, gaining a huge scope of bio‐melatonin for pharmaceutical industries [8]. Not only in mammals but over the past two decades, endogenous melatonin in plants has experienced significant importance [12, 13, 14]. According to the endosymbiotic theory, phagocytosis between bacteria and primitive eukaryotes evolved into mitochondria and chloroplasts, which are responsible for melatonin in existing plant species [15, 16]. However, the supply of raw materials from plant origins could limit the production of adequate melatonin [17]. Moreover, the exogenous melatonin application as a biostimulant modulates multiple physiological responses in plants' growth and development when thriving under stressful conditions [18, 19, 20, 21, 22]. Nevertheless, the ongoing debate about the presence and role of melatonin in mammals and plants continues to spark interest, while their biosynthetic pathways are better understood than in microorganisms. New possibilities will emerge in bio‐melatonin research through comprehensive knowledge of melatonin biosynthesis in melatonin‐producing microorganisms (M‐PMs).

Plant growth‐promoting microorganisms (PGPMs) now function as essential biological control agents by using direct or indirect symbiotic relationships for eco‐friendly plant health management. PGPMs activate defensive mechanisms that help plants withstand different types of abiotic and biotic stresses [23, 24, 25, 26]. Similar to PGPMs, bio‐melatonin derived from M‐PMs may offer an eco‐friendly alternative to synthetic melatonin for crop management. In this context, recent studies suggest that melatonin may function as a molecule for the transmission of bidirectional information between the plant and soil microorganisms (rhizobiome) [27]. Therefore, this review presents a broad overview of melatonin biosynthesis pathways in yeast, algae/plants, fungi, bacteria, and *Escherichia coli*. In addition, we focus on how microbial melatonin mediates plant‐microbe interactions under stressful environments.

## 2 Melatonin Biosynthesis in Microorganisms

**Figure 1:**
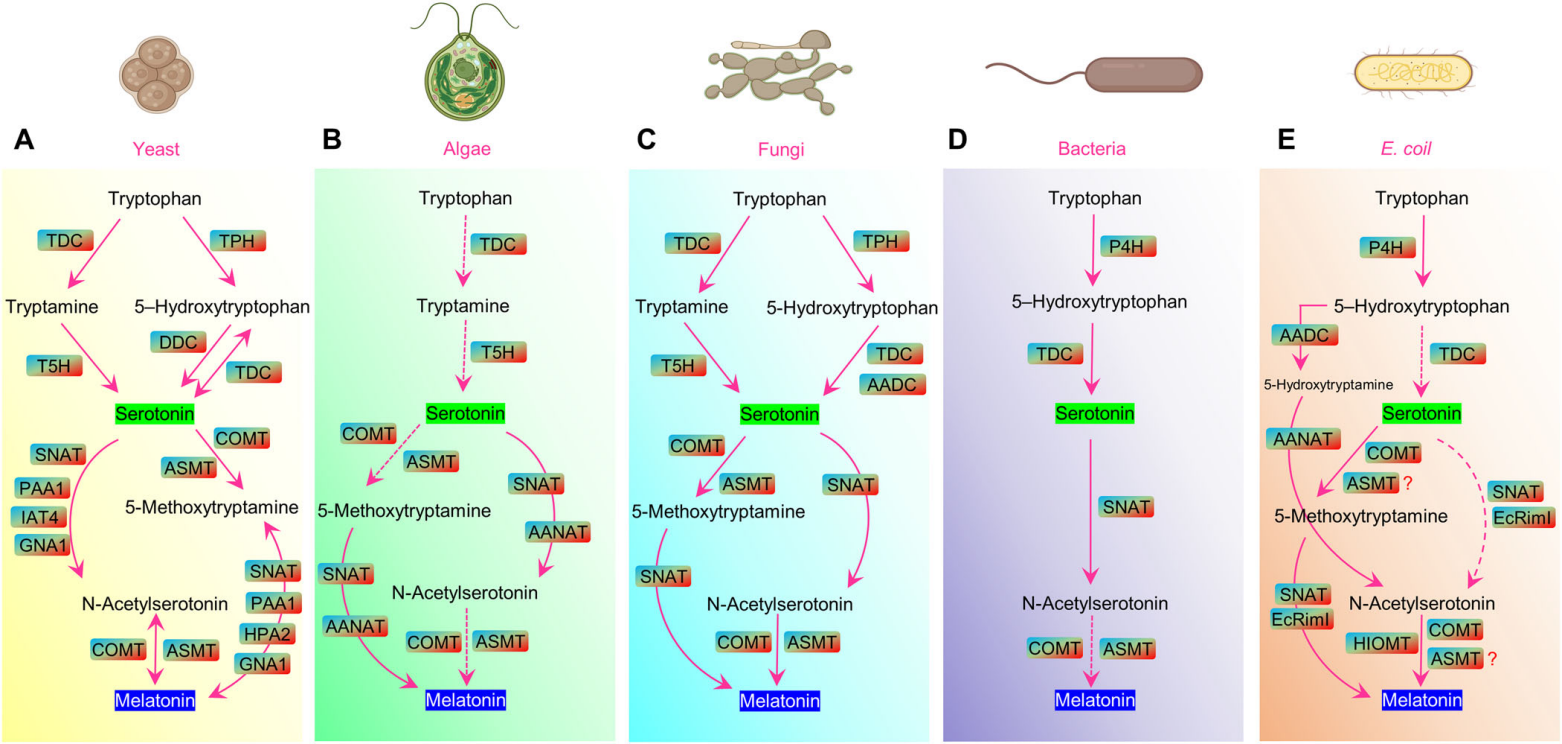
The figure illustrates the multi‐step enzymatic pathway for melatonin synthesis in microorganisms (Yeast, algae, fungi, bacteria, and *E. coil*). It details the key enzymes involved in the sequential conversion of tryptophan to melatonin. tryptophan decarboxylase (TDC), tryptophan hydroxylase (TPH), and tryptamine 5‐ hydroxylase (T5H), phenylalanine 4‐hydroxylase (P4H), 5‐hydroxy‐l‐tryptophan decarboxylase (DDC), aromatic l‐amino acid decarboxylase (AADC), caffeic acid O‐methyltransferase (COMT), *Escherichia coil* RimI (EcRimI), N‐acetylserotonin methyltransferase (ASMT), serotonin N‐acetyltransferase (SNAT), arylalkylamine N‐acetyltransferase (AANAT), PAA1 (polyamine acetyltransferase), HPA2 (histone acetyltransferase), GNA1 (glucosamine‐6‐phosphate N‐acetyltransferase), and hydroxyindole‐O‐methyltransferase (HIOMT). Both dashed lines and questioned marks present unidentified enzymes and pathways.

l‐tryptophan served as the initial component to synthesize serotonin, which proceeded into melatonin through a sequential action of enzymes and additional substrates. Since the discovery of melatonin in the bovine pineal gland, extensive research has been conducted to elucidate its biosynthesis in mammals and in plants [13, 16, 28, 29]. Despite substantial progress in understanding melatonin biosynthesis in animals and plants, the biosynthesis pathway of microorganisms remains an ongoing investigation [30]. A review conducted by Arnao et al. [8] focuses on commercial strategies for obtaining bio‐melatonin and also compares melatonin biosynthesis among animals and plants alongside yeast and algae, yet fails to provide details about microbial melatonin biosynthesis processes. Moreover, there is an inherent limitation in existing knowledge of the biosynthesis and production of microbial melatonin due to insufficient experimental studies, which hinders a critical discussion of melatonin biosynthesis. This knowledge gap is further compounded by the significant challenge of identifying new M‐PM strains. From the billions of microbes in nature, the process of selecting a single strain that not only produces a higher level of melatonin but also has a fully characterized biosynthetic pathway is often difficult due to their low melatonin production and lack of cost‐effective screening methods. However, we aim to understand the current state of known pathways of melatonin production in various microbiota, including yeast, algae, fungi, bacteria, and *E. coli*. Simultaneously, we discuss the existing knowledge gaps. Table 1 and Figure 1 describe the observed differences in melatonin production and the enzymes involved in its biosynthesis based on current knowledge of yeast, algae, fungi, bacteria, and *E. coli*.

**Table 1:** Endogenous melatonin levels in different studied microorganisms.

Type of microorganisms	Scientific name name	Melatonin level	Enzymes	References
Yeast	*Saccharomyces cerevisiae*	9.3–94.7 ng/mg protein	—	Sprenger et al. [5]
	*S. cerevisiae* (ARM, and QA23)	Detected but not quantified	—	Rodriguez‐Naranjo et al. [31]
	*S. uvarum* (Lalvin S6U)			
	*S. cerevisiae* var. bayanus (Uvaferm BC)			
	*Saccharomyces cerevisiae* UMY255	Detected but not quantified	—	Vigentini et al. [32]
	*S. cerevisiae* EC1118,			
	*S. cerevisiae* IOC18–2007			
	*Torulaspora delbrueckii* UMY196			
	*T. delbrueckii* UMY336			
	*T. delbrueckii* CBS1146T			
	*Zygosaccharomyces bailii* ATCC36947			
	*S. cerevisiae* SCE‐iL3‐HM‐40, 41, 42, and 43	0.04–1.93 mg/L	TPH, PTS, SPR, PCBD1, DHPR, DDC, AANAT, and ASMT	Germann et al. [33]
	*Saccharomyces cerevisiae*	0.4–2.4 ng/L	—	Fernández‐Cruz et al. [34]
	(Red Fruit, ES488, and Lalvin QA23)	1.0 ng/L		
		0.8–0.9 ng/L		
	*Torulaspora delbrueckii*			
	*Metschnikowia pulcherrima*			
	*S. cerevisiae* P24	60.83 pg 10^9^ cells	—	Fernandez‐Cruz et al. [35]
	*S. cerevisiae* QA23	0.0079–85.8813 ng/mL	TDC, T5H, DDC, SNAT, COMT, and ASMT	Muñiz‐Calvo et al. [36]
	*Hanseniaspora uvarum*‐Y1‐4	0.16–1.05 ng/mL	—	Jiao et al. [37]
	*S. cerevisiae*‐Y1‐4	0.24–0.77 ng/mL		
	*H. uvarum*‐P1‐4	0.05–0.63 ng/mL		
	*S. cerevisiae*‐P1‐4	0.13–0.58 ng/mL		
	*S. cerevisiae* P24	ND	—	Kung et al. [38]
Algae	*Gonyaulax polyedra*	0.16–1.00 Cynst/total cells	—	Balzer, Hardeland [39]
	*G. polyedra*	0.2–2.3 ng/mg protein approx.	—	Poeggeler et al. [40], Poeggeler, Hardeland [41]
	*Pterygophora californica*	16.2 ng/mL	—	Fuhrberg et al. [42]
	*Ulva sp*	0.5 ng/mL	—	Tal et al. [43]
		17 ng/g FW under Cd stress		
	*Chlamydomonas reinhardtii* CC‐1690	45.6 ng/g of cell pellet	—	Tal et al. [44]
	*Pyropia yezoensis*	0.16–0.23 ng/g FW	SNAT	Byeon et al. [45]
	*C. reinhardtii*	73.3 pg/mL	AANAT	Hwang, Back [46], Zhang et al. [47]
Fungi	*Trichoderma koningii*	18.681 µg/g DW	—	[48]
	*T. harzianum*	15.096 µg/g DW		
	*T. asperellum*	27.588 µg/g DW		
	*T. longibrachiatum*	13.863 µg/g DW		
	*T. viride*	11.555 µg/g DW		
	*Volvariella volvacea*	12–28 ng/g FW under control approx.	TDC, T5H, SNAT, COMT, and ASMT	Gao et al. [49]
		12–30 ng/g FW under heavy metal approx.		
	*Tolypocladium guangdongense* CCTCCM206051	0.4–18 ng/g FW approx.	AADC, TDC1, TDC2. TPH, T5H, SNAT1, SNAT2, ASMT1, and ASMT2	Wang et al. [50]
	*Exophiala pisciphila*	120–180 ng/g FW under control approx.	TDC, SNAT, and ASMT	Yu et al. [51]
		50–370 ng/g FW under heavy metals approx.		
	*Neurospora crassa*	NM	GPR‐3	Maienza et al. [52]
Bacteria	*Erythrobacter longus*	29.2 ng/mg protein at 120 min	—	Tilden et al. [53]
	*Synechocystis* sp. PCC 6803	ND	SNAT	Byeon et al. [54]
	*Bacillus amyloliquefaciens* SB‐9	0.87 ng/mL	TDC and SNAT	Jiao et al. [55]
	*B. thuringiensis* CS‐9	0.53 ng/mL		
	*Agrobacterium tumefaciens* CS‐30	0.22 ng/mL		
	*Pseudomonas fluorescens* RG11	1.32 ng/mL	P4H	Ma et al. [56], Jiao et al. [55]
	*Enterobacter* 64S1	99.0 ng/mL	—	Jofre et al. [27]
	*Pseudomonas* 42P4	402.9 ng/mL		
	*Bacillus safensis* EH143	0.2 ng/mL under control approx.	—	Kwon et al. [57]
		0.2–17 ng/mL under stress approx.		
*E. coli*	*E. coli*	~40 ng/mL	SNAT and COMT	Byeon, Back [58]
	*E. coli* BW25113∆tnaA	12.29–18.64 ng/mL	P4H and COMT	Zhang et al. [59]
	*E. coli* RimI	301–707 ng/mL	SNAT/EcRimI	Lee, Back [60]

Abbreviations: DW, dry weight; FW, fresh weight; ND, not detected; NM, not mentioned.

### 2.1 Yeast

The presence of melatonin in food and beverages is taken seriously when its health benefits are esteemed [31]. In particular, either wine or beer is consumed globally and holds a variety of bioactive phytochemicals and nutraceutical compounds [32, 61]. Yeast is a single‐celled eukaryotic microorganism that participates in the fermentation process of wine and beer [62]. During the fermentation process, an increased melatonin level was noticed, which sparked the understanding of melatonin biosynthesis in yeast [5, 31, 32, 34, 61]. *Saccharomyces cerevisiae* has been widely used to characterize the melatonin biosynthetic pathway in yeast. Numerous studies found a significant amount of endogenous melatonin in *S. cerevisiae* or non‐*Saccharomyces* strains used in the fermentation of wine and beer from cereals and grapes during alcoholic fermentation (Table 1) [31, 32, 34, 61]. Recently, Jiao et al. [37] identified thirteen (13) yeast species involved in melatonin synthesis, revealing significant variations in melatonin levels among fermented samples associated with different grape plant‐surface microbial communities. The significant variation in melatonin production among different strains of the same species needs to be revisited to justify this discrepancy.

Parallel to the identification of melatonin levels, it is important to understand the specific enzymes and substrates that regulate the final melatonin production in yeast. The biosynthesis of melatonin from *S. cerevisiae* was meticulously researched by Germann et al. [33] and Muñiz‐Calvo et al. [36]. We have illustrated melatonin synthesis in *S. cerevisiae* following a combined analysis of the two study results (Figure 1A). *S. cerevisiae* initiates melatonin biosynthesis by modifying l‐tryptophan into two separate compounds, which include tryptamine [36] and 5‐hydroxytryptophan [33]. In contrast, animals primarily convert l‐tryptophan into 5‐hydroxytryptophan through the action of tryptophan hydroxylase (TPH) [16]. The conversion of 5‐hydroxytryptophan from l‐tryptophan in the *S. cerevisiae* strain seems elusive based on the studies by Muñiz‐Calvo et al. [36]; instead, tryptophan decarboxylase (TDC) is responsible for the decarboxylation to tryptamine. In contrast, Muñiz‐Calvo et al. [36] reported a reversible reaction in which 5‐hydroxytryptophan is converted to serotonin, facilitated by both 5‐hydroxy‐l‐tryptophan decarboxylase (DDC) and TDC. An initial study by Park et al. [63] also tested that rice‐expressed TDC showed an accumulation of serotonin from 5‐hydroxytryptophan. However, the discrepancies in the conversion of l‐tryptophan into 5‐hydroxytryptophan in yeast still cannot be overlooked when a recent study in *S. cerevisiae* STG S101 strain found successful production of 5‐hydroxytryptophan [38]. After 5‐hydroxytryptophan, serotonin serves as an intermediate precursor for two key intermediates: 5‐methoxytryptamine and N‐acetylserotonin. These conversions are mediated by the enzymes serotonin N‐acetyltransferase (SNAT), caffeic acid O‐methyltransferase (COMT), and N‐acetylserotonin methyltransferase (ASMT), which complete the final steps of melatonin biosynthesis in *S. cerevisiae* (Figure 1A). Despite these proposed routes based on the isotope labeling approaches, three functionally associated melatonin biosynthetic enzymes in *S. cerevisiae*, PAA1 (polyamine acetyltransferase), HPA2 (histone acetyltransferase), IAT4 (putative indolamine N‐acetyltransferase), and GNA1 (glucosamine‐6‐phosphate N‐acetyltransferase), were identified from SNAT homologs [64, 65, 66]. While PAA1 and GNA1 can acetylate both serotonin and 5‐methoxytryptamine, HAP2 and IAT4 showed activity towards 5‐methoxytryptamine and serotonin only, respectively (Figure 1A). These abovementioned outcomes indicate the ongoing progress made in melatonin production in yeast.

### 2.2 Algae

The first identification of melatonin in the unicellular photoautotrophic algae was *Gonyaulax ployedra* [39, 40, 41]. Melatonin is also detected in a multicellular alga *Pterygophora californica*, triggered by both low temperature and the onset of darkness [42]. The pathway that leads to melatonin biosynthesis in photosynthetic algae remains elusive despite the established understanding of plant melatonin production [43]. Tal et al. [43] utilized a quick and simple methodology to estimate the algal melatonin levels using a marine macroalga *Ulva sp*. (Table 1).

To date in algae, only two key enzymes involved in melatonin biosynthesis, SNAT and AANAT, have been studied in *Pyropia yezoensis* [45] and *Chlamydomonas reinhardtii* [46, 67], respectively (Figure 1B). These enzymes are essential for the conversion of serotonin into melatonin. An overexpressed *AANAT* in *C. reinhardtii* showed enhanced melatonin levels under salt stress [67]. Notably, *CrAANAT*, a homolog of AANAT, which is typically found in mammals, has been detected in the genome of *C. reinhardtii* but is unknown in other green algae and higher plants [46, 68, 69, 70]. Therefore, the lack of studies limits our ability to conduct an in‐depth discussion on melatonin production in algae. This limitation could be addressed through continuous efforts to identify melatonin in various algal species, which may add an attractive value to these photosynthetic organisms, which already hold significant global economic importance.

### 2.3 Fungi

Similar to the melatonin production in unicellular fungi of *S. cerevisiae*, many more fungi are reported to produce endogenous melatonin, including saprophytic soil fungi, Arbuscular mycorrhizal fungi, and endophytic fungi [71, 72]. Several *Trichoderma spp*. were used to test whether they exist intercellular melatonin levels using liquid chromatography‐tandem mass spectrometry [48]. Melatonin levels were recorded between 11.5 and 27.5 µg/g dry mass, which was exceeded more under different stress conditions (Table 1). Moreover, in *Volvariella volvacea* and *Exophiala pisciphila*, melatonin contents were increased under control and heavy metal conditions [49, 51]. Studies on melatonin biosynthetic gene expressions are performed to understand the pathways. Under control and stress conditions, *TDC*, *T5H*, *SNAT*, *COMT*, and *ASMT* were expressed in *V. volvacea*, and further melatonin supplementation triggered their expressions (Figure 1C and Table 1) [49]. Wang et al. (2021) also demonstrated the intercellular melatonin synthesis pathways in an edible fungus, *Tolypocladium guangdongense*. Increased melatonin and its intermediate levels were found in the mycelium, primordial, and fruiting body stages of *T. guangdongense* under Congo red (CR), cold, and heat stresses. Relative mRNA expression of key enzymes of melatonin biosynthesis in *T. guangdongense* shares the same biosynthetic pathway as yeast (Figure 1C). Moreover, gene expression analysis of *EpSNAT1* and *EpASMT1* in *E. pisciphila* later enhanced our understanding of the melatonin biosynthesis pathway (Figure 1C) [51].

Melatonin receptors, including MT_1_ and MT_2_, from the family of G protein‐coupled receptors (GPCRs) can target melatonin by expressing in the mammal central nervous system and peripheral tissues [73]. This discovery provides possibilities for drug design in neurohormonal regulation, where melatonin receptors are coupled with the G protein. Recently, a new melatonin receptor, GPR‐3, has been identified in a model fungal organism, *Neurospora crassa*, employing the structural similarity assessment through AlphaFold2 and Dali [52]. Four fungal receptors of GPCRs (GPR‐1‐4) were initially tested, and later GPR‐3 was used as a selective melatonin‐related receptor in *N. crassa*, presenting as a future model organism for the melatonin signaling pathways. A reduced melatonin level was noticed in *gpr‐3* knockouts compared to the wild type, supporting the involvement of GPR‐3 in melatonin receptors in *N. crassa*. However, the total characterization of GPR‐3 is still a challenge in *N. crassa* to confirm the authenticity with MT1/MT2, but would be the addition of comprehensive knowledge of melatonin production in fungi.

### 2.4 Bacteria

Earlier studies by Manchester et al. [74] and [53] showed the endogenous melatonin level in the photosynthetic prokaryote, *Rhodospirillum rubrum* and *Erythrobacter longus* under continuous darkness or continuous light (Table 1). Higher melatonin level was recorded in the dark than in light conditions. After a decade, molecular characterization of the SNAT enzyme has been employed in the cyanobacterium *Synechocystis sp*. PCC 6803 [54]. Primarily, the rice *SNAT* gene was used to screen the homology in this bacterium, and an identified and expressed cyanobacterium *SNAT‐like gene* (*cSNAT*) in *E. coli*. *cSNAT* was markedly conserved in other cyanobacterial taxa and seems to be thermotolerant.

Later, the involvement of phenylalanine 4‐hydroxylase (P4H) was identified in the synthesis of 5‐hydroxytryptophan [75]. In vitro analysis showed that the purified P4H protein of *Pseudomonas fluorescens* converts l‐tryptophan to 5‐hydroxytryptophan, whereas animals rely on TPH for this conversion (Figure 1D). Notably, during in vitro incubation, tryptamine was not detected in *P. fluorescens* [56], where tryptamine was converted by TDC in yeast and fungi. In *P. fluorescens*, TDC synthesizes 5‐hydroxytryptophan into serotonin, which serves as a vital intermediate substance in melatonin production (Figure 1D). Moreover, a study by Jofre et al. [27] demonstrated that *Pseudomonas* 42P4 and *Enterobacter* 64S1 synthesized melatonin together with indole‐3‐acetic acid through a chemically defined medium that utilized tryptophan as a precursor. However, no genetic analysis was carried out to identify the enzymes that produce melatonin in their bodies.

### 2.5 E. coli

Melatonin synthesis in *E. coli* begins with l‐tryptophan, which is converted to 5‐hydroxytryptophan by P4H Zhang et al. [59] and Wang et al. [76]. During the biosynthesis of melatonin, most organisms rely on TPH to catalyze 5‐hydroxytryptophan, but *E. coli* lacks this enzyme, and even the TPH gene in the biosynthetic cluster was not explicitly mentioned in the previous study [59]. Additionally, TDC has not been detected in *E. coli*, which is responsible for converting 5‐hydroxytryptophan to serotonin (Figure 1E). Instead, Wang et al. [76] reported that AADC facilitates the conversion of 5‐hydroxytryptophan into 5‐hydroxytryptamine, followed by arylalkylamine N‐acetyltransferase (AANAT) catalyzing the synthesis of N‐acetylserotonin. The predominant N‐acetylserotonin synthesis from serotonin in plants and animals has also been identified in *E. coli*. Byeon et al. [58] first characterized the SNAT genes in recombinant *E. coli*, which facilitate melatonin production by converting serotonin. Lee et al. [60] also demonstrated that the overexpression of *EcRimI*, a potential SNAT ortholog in *E. coli*, resulted in increased melatonin production compared to the control strain in the presence of 5‐methoxytryptamine. The results show that *EcRimI* seems to participate in serotonin to N‐acetylserotonin conversion (Figure 1E). The COMT gene was also identified in *E. coli* by Byeon, Back [58], which completes the melatonin production from N‐acetylserotonin. Moreover, Zhang et al. [59] and Wang et al. [76] have characterized COMT and hydroxyindole‐O‐methyltransferase (HIOMT), providing more insights into melatonin production in *E. coli*. However, ASMT, a primary enzyme in melatonin biosynthesis, has yet to be identified in *E. coil*. Byeon, Back [58] failed to identify ASMT due to low enzyme activity in *E. coli*. Overall, the ongoing research on melatonin biosynthesis in *E. coli* continues to expand for discoveries about enzymatic regulation.

## 3 Melatonin‐Producing Microorganisms Contribute to Both Self‐Defense and Plant Protection

Given the emerging evidence of melatonin production from microorganisms, this section proposes a unifying hypothesis that M‐PMs confer resilience through a two‐tiered mechanism: (1) enhancing their survival by activating endogenous redox and stress‐response pathways; (2) delivering or stimulating melatonin‐dependent defense responses in associated plants, thereby supporting plant growth and stress response.

Melatonin production in yeast is discussed in the previous section, but how endogenous melatonin in these species contributes to their self‐defense in adverse conditions is also still unknown (the effect of exogenous melatonin in yeast is discussed in the next section). Furthermore, the importance of yeast in sustainable agriculture is well‐reviewed by Mukherjee et al. [77], as it promotes plant growth through hormone secretions, including ACC deaminase, cytokinin, indole‐3‐acetic acid, indole‐3‐pyruvic acid, and zeatin, as well as inhibiting soil‐borne pathogen *Rhizoctonia solani*, and many more benefits. These findings suggest that M‐P‐yeast needs to be challenged in agricultural applications, which has essentially been denied until today.

**Figure 2:**
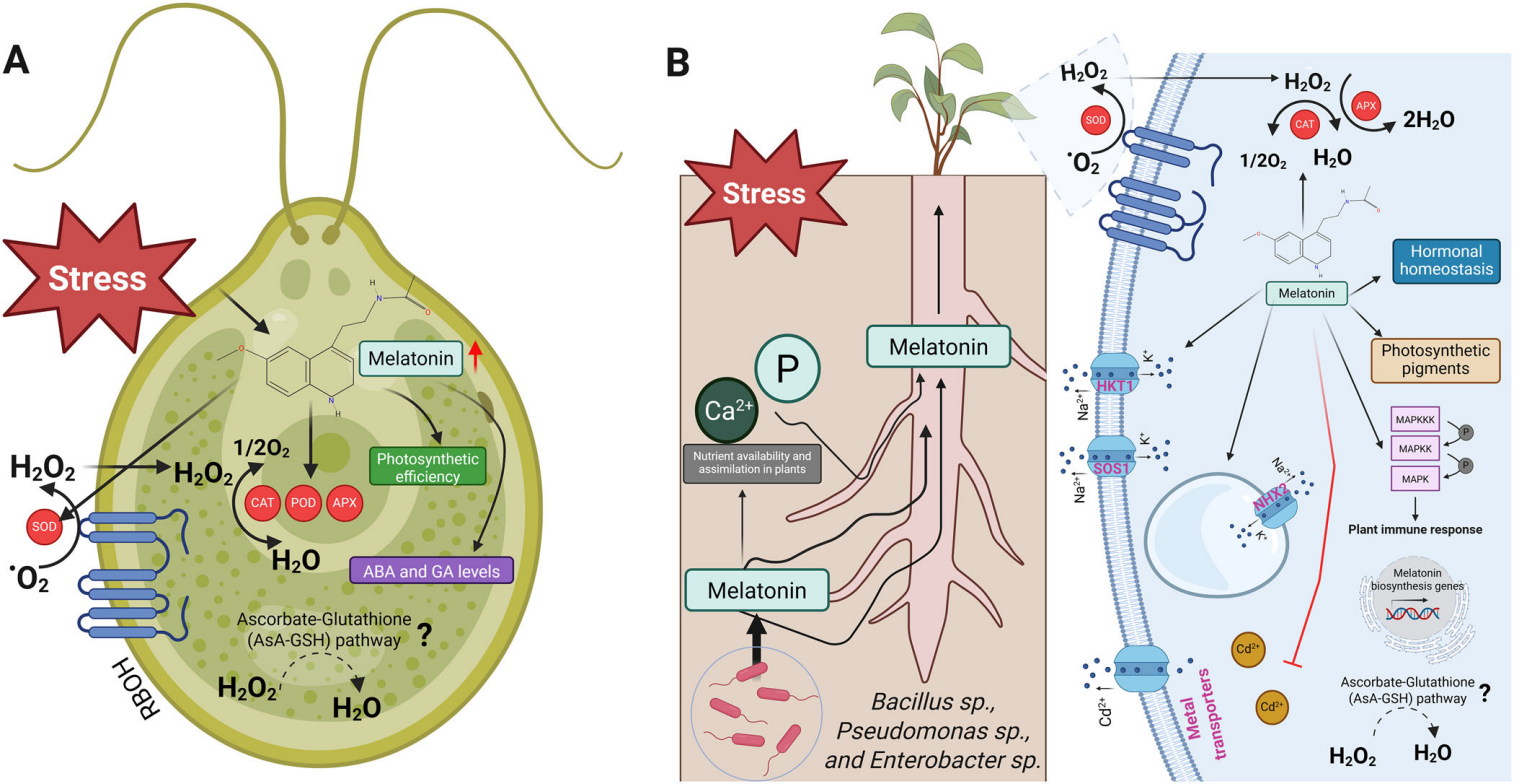
Microbial melatonin production likely contributes to stress resilience in both microbes and their hosts. (A) Under environmental stress conditions, algae produce excessive ROS, which impairs cellular stability. However, melatonin‐producing algae increase the antioxidant activities that balance ROS levels to counteract cellular damage. This process also enhances photosynthetic efficiency and hormonal balance. (B) The application of M‐PMs to the rhizosphere elevates the melatonin biosynthetic enzymes and endogenous melatonin levels in host plants through a symbiotic relationship that facilitates melatonin exchange. Similar to microbes, elevated melatonin in plants helps mitigate ROS levels under stress conditions by magnifying the antioxidant activities. Notably, under salt stress, the elevated melatonin in the host improves the K^+^/Na^+^ ratio by upregulating Na+ transporters, which is critical under salt stress. Consistently, the upregulation of metal transporters has been noticed in host plants, which helps to reduce the Cd^2+^ accumulation. Additionally, M‐PMs contribute to improved Ca^2+^ and P availability and uptake in plants, potentially supporting stress resilience. The increased melatonin levels in the host plant are also involved in MAPK‐mediated plant bacterial resistance. These tolerance mechanisms collectively promote plant photosynthesis, growth, and development. Although the nonenzymatic ascorbate‐glutathione‐based ROS detoxification is important, its role in M‐PMs‐mediated stress response in both microbes and plants has yet to be investigated.

Under unfavorable environments, organisms maintain a delicate balance between the level of reactive oxygen species (ROS) and the antioxidant capacities to scavenge free radicals to minimize cell damage [78]. Similarly, melatonin acts as a potent antioxidant itself as well as modulates enzymatic or nonenzymatic antioxidant properties that help protect from oxidative damage in mammals and plants [21, 79, 80, 81]. Together with higher organisms, melatonin production in microorganisms protects them from abiotic stresses by increasing the defense metabolisms. For instance, studies on both macroalgae (*Ulva* sp.) [43] and microalgae (*C. reinhardtii*) [44] highlights its ubiquitous support for maintaining redox homeostasis during photosynthesis (Figure 2A), which is also indispensable in plants. Melatonin production in genetically engineered *C. reinhardtii* helps in enhancing catalase (CAT), superoxide dismutase (SOD), and peroxidase (POD) activity by reducing the malondialdehyde (MDA) level under salt stress [67]. This improved antioxidant activities in the overexpression of the *AANAT* gene of *C. reinhardtii* markedly protect the integrity of the cell membrane, which could help alga cells limit salt stress and mitigate oxidative damage (Figure 2A). In addition, a homolog of *C. reinhardtii* AANAT (*CrAANAT*) expressed in the rice plant increased in elevated melatonin levels, increased leaf angle, and enhanced seed length [46]. Together, these results support our hypothesis that microbially derived melatonin acts as a systemic stress‐mitigating agent, not only protecting algae themselves but also offering exchangeable benefits to host plants.

M‐P‐fungi possess defense systems that help them build resistance against various abiotic stresses. *T. guangdongense* is considered an invaluable edible mushroom, and its increased intercellular melatonin levels through Congo red (CR) stress, together with heat and cold stress, demonstrate stress protection capabilities [50]. Moreover, when subjected to CdCl_2_, CuSO_4_, and H_2_O_2_, *Trichoderma asperellum* exhibited a noteworthy production of melatonin than the control [48]. *Exophiala pisciphila* demonstrated a reduction in MDA and free radical levels by augmenting SOD levels in the presence of various heavy metals [51]. These findings indicate that M‐P‐fungal species are also placed into our hypothesis that enables them to protect under adverse climatic conditions, which is mainly a melatonin‐mediated defense. Nonetheless, the utilization of M‐P‐fungi as plant‐growth‐promoting fungi (PGPF) has yet to be explored to understand their rhizospheric interaction with host plants.

On the other hand, studies have proven that supplemental melatonin affects plant‐pathogenic fungi, which represent serious biotic hazards to plant development and yield production [82]. For instance, pre‐treating apple trees with 0.1 and 0.5 mM melatonin helped them fight off Marssonina apple blotch (*Diplocarpon mali*) [83]. Similarly, applying 100 μmol L^−1^ melatonin to banana plants made them more resistant to Fusarium wilt [84]. RNA‐seq was employed in *Lilium leaves* supplemented with 2 mM melatonin enhanced the defense‐related differentially expressed genes and mitogen‐activated protein kinases (MAPK) signaling in *B. elliptica*−infected leaves [85]. RT–qPCR results corroborated the RNA‐seq results, where the exogenous melatonin and *B. elliptica* infection significantly augmented the relative expression of MEKK1, MKK4/5, MKK2, and MPK3. In watermelon, either melatonin supplementation or transgenic *SANT OE* lines regulated downstream signaling cascades, including MAPK, WRKY, and serine/threonine kinase [86], along with increased plant hormone levels that withstand the *Podosphaera xanthii* and *Phytophthora capsici*. Many more studies reported that exogenous melatonin application limits the fungal infestation in vegetables and could contribute to minimizing global crop loss [87, 88, 89, 90]. Not only in fruits, flowers, and vegetables, the application of melatonin enhances fungal resistance in commercial crops. Melatonin (0.1 mM) treatments reduced *F. oxysporum* and *Penicillium brevicompactum* infestation in ginger rhizome during postharvest periods through the upregulation of defense‐related gene expressions and enzyme activities [91]. Cotton immunity was compromised in *GhSNAT1*‐ and *GhCOMT*‐suppressed plants but was restored upon melatonin application [92]. Together with biotic stress, melatonin application influences microbial activities, leading to abiotic stress. In drought stress, the co‐application of melatonin (0.2 mM) and arbuscular mycorrhizal fungi significantly promoted tobacco seedling growth while mitigating the adverse effects of stress [93]. Furthermore, melatonin (0.09 mM) and fungal metabolite applications mitigate cadmium toxicity in tomato plants by modulating endogenous phytohormone levels (jasmonic acid and salicylic acid) [94]. Although multiple benefits have been observed by exogenous application of melatonin, it does not fully clarify the specific roles of fungi with melatonin‐producing potential.

The symbiotic relationship between rhizobacteria and plants is vital for the mutual transfer of phytohormones and bioactive secondary metabolites (Figure 2B). Current research on M‐P‐bacteria and their effects on plant physiology remains in its early stage, while plant‐growth‐promoting rhizobacteria (PGPR) have received extensive attention for their symbiotic interactions with plants [95, 96]. The present research into M‐P‐bacteria's ability to improve plant development and defense against stress shows limited progress, thus suggesting more study is required in this emerging field. *Bacillus amyloliquefaciens* SB9 demonstrates high endogenous melatonin production [55]. The application of *B. amyloliquefaciens* SB9 to grapevine plant root zones results in effective melatonin transfer to the host which subsequently promotes the activation of melatonin biosynthesis genes (*VvTDC1*, *VvTDC2*, *VvTDC3*, and *VvSNAT*) and increases melatonin levels in plant roots [55]. Melatonin elevation contents in plant roots later help in ROS alleviation under salt and drought stress conditions, pointing out its potential role in stress mitigation and plant defense (Figure 2B). Furthermore, Jofre et al. [27] reported two PGPR strains: *Pseudomonas* 42P4 and *Enterobacter* 64S1 which showed elevated amounts of endogenous melatonin. Upon inoculation of these two M‐P‐bacteria to Arabidopsis plants under drought stress, enhanced plant biomass was noticed with minimal oxidative damage as an estimated lower level of MDA (Figure 2B). Similarly, another M‐P‐bacteria, *B. safensis* EH143 and EH151, protected the soybean plants from salt and cadmium stress [97, 98]. Application of *B. safensis* EH143 and EH151 produces and shares melatonin with the host plant in the rhizosphere; hence, the elevation of endogenous melatonin in plants regulates the K^+^/Na^+^ ratio to combat salt stress (Figure 2B). Moreover, inoculation with both strains enhanced Ca^2+^ and P accumulation in soybean plants, suggesting that M‐PMs may influence soil nutrient availability and their assimilation in plants (Figure 2B). In particular, M‐PMs‐dependent Ca^2+^ may act as a secondary messenger to mitigate the adverse effects of stress [99, 100]. Future studies on macro‐ and micronutrient uptake will enrich our knowledge of overall nutrient availability and acquisition by the host in response to M‐PMs application. Furthermore, the ability to inhibit toxic ion uptake by soybean plants associated with transcriptional and hormonal modulation in *B. safensis* EH143 and EH151 could be considered an innovative PGPR with melatonin production ability. These findings lend preliminary support to our hypothesis that bacterial melatonin can be functionally exchanged with host plants, facilitating their stress resilience. However, the mechanisms of bacterial melatonin exchange with host plants are being ignored continuously and remain key gaps in our understanding. To date, we have knowledge that hosts and microbes belong to each other wholly or partially to support each other, but how host pick their rhizospheric friends is still enigmatic. To uncover the mechanisms, we may need to understand the perception of M‐PMs by the host. A recent study reflects this perception theory that a key plant immunity regulator in the soybean plant, RIN4, is phosphorylated just after contact with rhizobia to enhance symbiosis [101]. Knowing such perception strategies between hosts and M‐PMs is challenging but crucial for knowing the tradeoff. Moreover, employing techniques like high‐throughput time‐lapse imaging [102] would allow for a visible, real‐time understanding of the living trade network between hosts and M‐PMs, which holds promise for unraveling the melatonin exchange process.

While M‐P‐bacteria show potential in modulating plant defense, much of our understanding comes from studies on either endogenous or exogenous melatonin in plants. In SNAT‐silenced Arabidopsis plants (*snat1* and *snat2*), melatonin levels dropped below 50%, displaying the susceptibility of *Pseudomonas syringae* pv. tomato DC3000 by compromising the salicylic acid (SA) and the defense genes, including *PR1*, *ICS1*, and *PDF1.2* [103]. In contrast, the addition of exogenous melatonin (10 and 20 µM) enhanced the resistance to *P. syringae* pv. tomato DC3000 in *Arabidopsis thaliana* through SA and nitric oxide (NO)‐dependent manner [104, 105]. These results emphasize the melatonin's role as a SA‐ and NO‐dependent upstream regulator for plant immunity. Similar to the previous discussion on fungal pathogens, melatonin is also required in MAPK signaling that activates innate immunity to confer bacterial resistance (Figure 2B) [106, 107]. Melatonin supplementation (20 µg mL^−1^) was found to regulate NO production and pathogenesis‐related proteins, *PR1b*, *PR8a*, and *PR9*, to protect rice plants from *Xanthomonas oryzae* pv. *oryzae* [108]. Furthermore, melatonin application (0.1 mM) alleviates the bacterial foodborne in cherry tomatoes [109] and angular leaf spots in cucumber plants [110]. The exogenous application of melatonin together with several bacterial species has also been studied to improve plant tolerance against various abiotic stresses [111, 112, 113, 114]. In the context of these findings, we understood that melatonin may play a central role in plant defense from bacterial disease.

Reports show *E. coli* has lower antioxidant content that results in higher ROS production levels under multiple stress conditions. Melatonin at low concentrations can potentially regulate redox homeostasis [115, 116, 117]. Enhanced melatonin production was noticed in *E. coli* expressing *SNAT* and *COMT* [58]. Thereby, the improved melatonin synthesis from useful microorganisms like *E. coli* plays multifaceted physiological roles. More specifically, the same research group later identified *EcRimI/SNAT* overexpression strain of *E. coli* that increased their protein N‐acetyltransferase activity which led to higher melatonin production under cadmium stress when compared to the control [60]. The enhanced growth of the *EcRimI/SNAT* overexpression strain under cadmium stress, demonstrating melatonin synthesis in *E. coli*, strengthens enzymatic activities and restrains the cadmium toxicity.

Together, these findings across diverse microbial taxa suggest that melatonin may represent a conserved, evolutionarily co‐opted molecule for stress mitigation, providing internal defense and potential cross‐species in plant‐microbe interactions, consistent with our proposed hypothesis. However, our observation is that beyond the M‐P‐bacteria, the application of the other M‐PMs remains largely uncharted from an agricultural perspective. The knowledge of M‐PMs' stability and their colonization in the rhizosphere remains unproven. Moreover, the cost‐effectiveness of M‐PMs compared to synthetic fertilizers is a major concern. Thus, these indicate that while the use of M‐PMs in agriculture holds promise, similar to PGPR, the concept is still in its preliminary stages and requires robust investigation for its broader agricultural application.

## 4 Exogenous Melatonin Application Enhances Microorganism Resistance to Stress Similarly to That of Plants

Earlier discussion explained the efficacy of intercellular melatonin in microorganisms enhances their resilience to stressful environments. Nonetheless, due to the limited accessibility of advanced techniques for detecting endogenous melatonin in microorganisms, the application of exogenous melatonin is steadily increasing. This exogenous application has been widely recognized for its efficacy as a powerful signaling molecule in plants. Melatonin with a lower concentration positively regulates plants' growth, development, and overall yield. Particularly, it has been shown to promote seed germination, shoot growth, root organogenesis, photosynthesis, floral transition, autophagy, anti‐senescence properties, and postharvest fruit ripening [18, 19, 118]. Melatonin controls these developmental processes by altering the levels of key plant hormones like auxin, cytokinin, gibberellic acid, abscisic acid, brassinosteroids, and ethylene [18, 19]. This hormonal modulation, along with changes in the transcriptional factors, potentially improves the plant physiology. Moreover, its protective role extends to plant biotechnology, where it aids in callus culture induction and plant biomass production by eliciting diverse secondary metabolites in in vitro cultures [14]. As previously mentioned, numerous studies have confirmed the positive roles of exogenous melatonin in mitigating abiotic and biotic stresses in plants.

Consistent with its effects in plants, exogenous melatonin also significantly impacts microbial growth and stress tolerance. For instance, under H_2_O_2_ stress, melatonin application on single‐celled and eukaryotic *S. cerevisiae* and non‐*S. cerevisiae* strains exhibited redox balance, fatty acid accumulation, and organic acid production to combat the ROS formation of these microorganisms and maintain their cell growth [119, 120, 121]. Furthermore, a comprehensive transcriptomic study was employed in *S. cerevisiae* under H_2_O_2_ exposure with or without melatonin supplementation [122], resulting in upregulation of ROS synthesis genes, water deprivation, protein folding, oxido‐reduction process, copper binding, zinc homeostasis, lipid metabolism, and many more. The downregulated genes are involved in mitochondrial organization and stability. Moreover, melatonin at low concentrations (1 µg mL^−1^) aids *S. cerevisiae* in coping with copper stress and accelerates fermentation but a high melatonin level (100 mg mL^−1^) produces an unpleasant smell that suppresses glucose metabolism and the fermentation process [123]. On the other hand, metabolomic analysis showed that moderate melatonin application increases copper resistance of *S. cerevisiae* by enhancing the synthesis of metallothionein (Cup1p), amino acids (methionine and cysteine), and N‐γ‐acetyl‐N‐2‐formyl‐5‐methoxykynurenamine (AFMK). These findings pinpoint that exogenous melatonin may act via redox‐sensitive transcription factors and posttranslational regulators, where direct molecular targets in melatonin‐dependent yeast bioengineering remain poorly defined.

In the previous section, we reviewed how intercellular melatonin levels enhanced algal metabolism to withstand several stressors. As microalgae have immense potential from a biotechnological perspective for food security [124], numerous studies have also pointed out how to protect the algal community from abiotic stresses or wastewater through exogenous melatonin application. Recently Zhao et al. [125] reviewed how melatonin integrates with algae to modulate metabolite levels, biomass, lipid, and carotenoid production. The reason why melatonin is applied during algal growth is primarily to enhance their resilience against external stresses. By regulating redox homeostasis, melatonin effectively controls ROS levels and restores photosynthetic efficiency, making its supplementation beneficial for algal species. Melatonin application (1 and 5 µM) in algal species (*C. reinhardtii*) improves photosystem II activity and cell growth, increases antioxidant activity, and enhances lipid accumulation; subsequently, it leads to elevated defense‐related gene expression, thereby reducing ROS levels which helps protect against cadmium, salt, and nitrogen stress [126, 127, 128]. Furthermore, in *Haematococcus pluvialis*, supplementing 10 to 20 µM of melatonin stimulates its biomass and lipid production while upregulating astaxanthin biosynthesis [15, 129, 130, 131, 132, 133]. Melatonin also modulates NO‐mediated MAPK signaling and cyclic adenosine monophosphate (cAMP) signaling pathways in *H. pluvialis* [129]. The addition of melatonin to *Monoraphidium* sp. enhances lipid content, ABA and GA levels, and autophagic processes, which increases the expression of the *atg8* gene family related to autophagy [130, 134, 135]. Based on these current outcomes, melatonin's role in algae seems to mirror its plant function, where melatonin supplementation regulates algal physiology and their resilience under stress conditions.

Parallel to algae, exogenous melatonin enhances fungal stress tolerance and self‐defense mechanisms. In *Volvariella volvacea*, treatment with 100 µM melatonin significantly reduces cellular damage [49]. In *Agaricus bisporus*, a highly nutritious and economically important edible mushroom, melatonin improves mitochondrial function by increasing the activities of complexes I and III, resulting in enhanced ATP production by reducing electron leakage driven by the upregulation of *AbNdufB9* and *AbRIP1* gene expression levels [136]. While the crosstalk of NO and melatonin regulates multiple mechanisms in plants, melatonin application (100 µM) also induces endogenous NO production in the bambusicolous fungus species (*Shiraia* sp. S9) [137]. The elevation of endogenous NO, modulates ROS levels, pinpointing the interplay between melatonin and NO in maintaining redox homeostasis in fungi [137], in a similar way to that of plants [18]. However, comparative and functional assays will be critical to a better understanding of melatonin‐NO interaction in fungi.

Collectively, examining the current concepts of the application of exogenous melatonin to microbes has been a key area of study for understanding its role in stress tolerance. We observed that stress tolerance efficacy in microbes varied within the species and diverse range of melatonin concentrations. However, a fundamental question remains: how do these high concentrations, typically applied exogenously, relate to the much lower levels of endogenous melatonin naturally produced by the microbes? When we compare these earlier data on microbial melatonin production displayed in Table 1, it is clear that studies often use significantly more melatonin in microbes than what is naturally present endogenously. Therefore, we must have a better understanding of both microbial and exogenous melatonin usage to develop sustainable and beneficial applications in agriculture.

## 5 Conclusions and Future Perspectives

Melatonin, either synthesized endogenously or applied exogenously, holds a fascinating importance in animals, plants, and microorganisms. Tremendous progress has been made in melatonin signaling and its roles in animals and plants, together with identifying the biosynthetic paths more accurately. In microorganisms, except yeast, none of the microorganisms have been studied deeply to characterize their melatonin biosynthesis routes. Based on the present studies, we noticed that microorganisms do not follow the same enzymes and substrates as either animals or plants for melatonin biosynthesis, which allows them to be distinguished. However, this review provides a glimpse of the beginning of the production process in microorganisms.

More importantly, numerous studies demonstrated beneficial effects of endogenous and exogenous melatonin on microbial physiology and plant‐microbe interactions. However, most of these findings are descriptive, and mechanistic studies linking melatonin perception and downstream signaling remain underexplored. In particular, how microbes supply melatonin to plants and whether it is actively transported or sensed, remains unresolved. Due to the scarce use of M‐PMs in agricultural studies, in‐depth conclusive remarks may need to be revisited. To advance this field, future research should be prioritized on (i) determining melatonin transport routes and modes of exchange between microbes and hosts; (ii) exploring the ecological relevance of M‐PMs during crop production. Overall, we believe M‐PMs have the potential to reshape rhizosphere melatonin ecology in future studies and bring a new path for sustainable agriculture.

## Author Contributions

**In‐Jung Lee**, **Marino B. Arnao**, **Byung‐Wook Yun**, and **Sang‐Mo Kang:** conceptualization. **Sang‐Mo Kang**, **Ashim Kumar Das**, and **Da‐SoL Lee:** data curation. **In‐Jung Lee**, **Marino B. Arnao**, and **Ashim Kumar Das:** writing – review and editing.

## Conflicts of Interest

The authors declare no conflicts of interest.

## Data Availability

The data that support the findings of this study are available on request from the corresponding author. The data are not publicly available due to privacy or ethical restrictions.
